# Expression and characterization of cecropinXJ, a bioactive antimicrobial peptide from *Bombyx mori* (Bombycidae, Lepidoptera) in *Escherichia coli*

**DOI:** 10.3892/etm.2013.1056

**Published:** 2013-04-09

**Authors:** LIJIE XIA, FUCHUN ZHANG, ZHONGYUAN LIU, JI MA, JIANHUA YANG

**Affiliations:** 1 Xinjiang Key Laboratory of Biological Resources and Genetic Engineering, College of Life Science and Technology, Xinjiang University, Urumqi, Xinjiang 830046, P.R. China;; 2 Department of Pediatrics, Section of Hematology/Oncology, Baylor College of Medicine, Houston, TX 77030, USA

**Keywords:** prokaryotic expression, antimicrobial peptide, antibacterial activity, antitumor activity

## Abstract

Insect antimicrobial peptides (AMPs) have a broad antimicrobial spectrum. To aid the characterization of the gene function and further applications, we cloned the gene of cecropinXJ into the prokaryotic expression vector pET32a and expressed cecropinXJ in *Escherichia coli* BL2l (DE3). Following induction by isopropyl-β-D-thiogalactoside (IPTG), a 25 kDa fusion peptide of cecropinXJ with a tagged thioredoxin (Trx) protein was highly expressed in *E. coli*. The yield was 10 mg/l culture medium following purification on nickel-nitrilotriacetic acid (Ni-NTA) metal affinity chromatography matrices. The purified recombinant antibacterial peptide, cecropinXJ, retained a high stability against *Staphylococcus aureus* over a temperature range from 4 to 100°C and a pH range from pH 2.0 to 12.0. The minimum inhibitory concentration (MIC) of the fusion protein against *S. aureus* was 0.4 *μ*M. The recombinant cecropinXJ is also cytotoxic to several types of human cancer cells.

## Introduction

Antimicrobial peptides (AMPs) are a class of immune-related peptides that provide the first line of defense to protect the host from invading pathogens (1). These peptides prevent bacterial infections and are particularly critical for invertebrates, such as insects, that lack lymphocytes and antibodies (2). AMPs have minimal toxicity and low sensitivity effects to the host (3). Thus, antimicrobial peptides may be applied in medicine, agriculture and food as new and safe antibiotics or antiseptic agents (4–7).

Cecropins, a group of small basic polypeptides mainly found in the hemolymph of insects, consist of 31–39 amino acid residues and have a broad spectrum, high heat stability and potent bacteriostatic activity (8–10). Eukaryotic cell expression or artificial synthesis of the cecropin gene is characterized by a low efficiency and high cost (11–13). In order to explore a cost-effective and scalable method for producing large quantities of active peptides, expression of recombinant peptides may be used. To date, various expression systems, including yeast (14), *E. coli* (15) and insect cells (16), have been established for the production of recombinant antibacterial proteins.

In recent years, studies concerning the expression of antimicrobial peptides have mainly focused on the use of fusion partners (17). Thioredoxin, a heat-stable and low molecular weight soluble protein in the prokaryotic cytoplasm, has been shown to display chaperone-like activity (18). Fusion proteins of antimicrobial peptides generated in *E. coli* reduce the toxic effect of antimicrobial peptides to the host cells and protect the small antimicrobial peptides from proteolytic degradation.

CecropinXJ, is a member of the cecropin family, which we first cloned from the larvae of the Xinjiang silkworm (*Bombyx mori*) by reverse transcription-polymerase chain reaction (RT-PCR) and 3′- and 5′- rapid amplification of cDNA ends (3′/5′-RACE). The complete amino acid sequence of the molecule was determined (19). In addition, we successfully expressed cecropinXJ in *Pichia pastoris*, where the expression levels were observed to be relatively low (20).

In the present study, we aimed to express cecropinXJ in a prokaryotic expression system, which combines the advantages of high expression levels, easy scale-up and inexpensive growth media. A vector derived from the commercial pET32a(+) was developed. This expression vector carrying the thioredoxin (Trx) gene and T7 promoter contained a 6xHis-tag to facilitate purification and enhance the expression yields of fusion proteins in *E. coli*. We constructed a recombinant expression vector pET32a-*cecropinXJ* and expressed recombinant cecropinXJ at high levels. A high yield of soluble recombinant cecropinXJ was obtained and purified. The purified recombinant cecropinXJ displayed strong antimicrobial activity to bacteria and fungi, as well as cytotoxicity to several types of human cancer cells.

## Material and methods

### Bacterial strains, vectors and enzymes

The prokaryotic plasmid pET32a(+) was purchased from Invitrogen (Beijing, China). The restriction enzymes, T4 DNA ligase, DNA ladder and pre-stained protein marker were purchased from Fermentas (Vilnius, Lithuania). *E. coli* DH (5α) and BL21(DE3) pLYsS competent cells were purchased from Takara (Dalian, China). PCR primers were synthesized by Shanghai Sangon Biological Engineering Technology & Services Co., Ltd. (Shanghai, China). Other reagents were obtained either from Sangon Chemical Reagent (Shanghai, China) or Sigma (St. Louis, MO, USA). The test microorganisms used in this study were obtained from the China General Microbiological Culture Collection Center (Beijing, China).

### Construction of recombinant pET32a-cecropinXJ expression vector

The sequence of the cecropinXJ gene was amplified and isolated from the plasmid pMD18-T-*cecropinXJ* (19), which carries the cDNA of *cecropinXJ* from the Xinjiang silkworm larvae (*Bombyx mori)*. The upstream primer Pl, 5′-CCG gaa ttc AGG TGG AAG ATC TTC AAG AAA ATT GAA AAA ATG GGC-3′ contained an *Eco*RI site (lower case), and the downstream primer P2, 5′-CCG ctc gag TCA TTT TCC TAT AGC TTT AGC CGA ACC AAG-3′ contained a *Xho*I site (lower case). The PCR reaction parameters were: pre-denaturation at 95°C for 1 min, denaturation at 95°C for 1 min, annealing at 95°C for 30 sec, extension at 55°C for 1 min for 30 cycles and 1 min extension at 72°C. The cecropinXJ gene and the vector pET32a plasmid were subjected to digestion with *Eco*RI and *Xho*I, and ligated using T4 DNA ligase. The recombinant pET32a-*cecropinXJ* was transformed into *E. coli* DH (5α) competent cells for amplification. Positive colonies resistant to ampicillin on a Luria-Bertani (LB) plate were selected and the plasmid pET32a-*cecropinXJ* was confirmed by restriction enzyme mapping and DNA sequencing.

### Expression of recombinant protein

The recombinant plasmid pET32a-*cecropinXJ* was transformed into *E. coli* BL21(DE3) competent cells for expression. The expression of the fusion protein was induced by the addition of 0.8 mM isopropyl-β-D-thiogalactoside (IPTG) once the optical density at 600 nm (OD_600_) of the culture had reached 0.6–0.8. After 5 h of induction, 1 ml culture was centrifuged at 8,000 × g for 5 min. The cell pellet was resuspended in 100 *μ*l phosphate-buffered saline (PBS) and analyzed by Tricine-SDS-PAGE. The expression level of recombinant cecropinXJ was determined by protein bands and quantified using densitometry (GeneTools software, Philomath, OR, USA). Bovine serum albumin (BSA) was used as a standard.

### Purification of recombinant protein

Following induction, 1 l culture was centrifuged at 8,000 × g for 5 min. The pellet was resuspended in 10 ml PBS and placed in an ice bath for ultrasonic lysis (200 W, 5 sec, 5 sec). The supernatant was further purification by 80% ammonium sulphate fractionation, dialysis and desalt, filtered using a 0.22 *μ*m filtration membrane and loaded onto a Ni-NTA agarose column (Qiagen, Hilden, Germany). Using the QIAexpressionist™ kit, the supernatant was mixed with equilibration buffer (50 mM sodium phosphate; pH 8.0; 0.3 M sodium chloride; and 10 mM imidazole) gently by agitation (150 rpm on a rotary shaker) at 4°C for 60 min. The column was washed with buffer (equilibration buffer) and the protein was eluted with elution buffer (50 mM sodium phosphate; pH 8.0; 0.3 M sodium chloride; and 250 mM imidazole). The eluate was concentrated through a 10 kDa cutoff Centriprep filter (Millipore, Bedford, CA, USA) for Tricine-SDS-PAGE and western blot analysis.

### Western blot analysis

The concentration of purified recombinant protein was detected using a Bradford protein assay kit. The supernatants were electrophoresed using 15% Tricine-SDS-PAGE and transferred onto a PVDF membrane (Pall Corporation, Washington, NY, USA). After being washed with TBST buffer (20 mM Tris-HCl, 150 mM NaCl and 0.05% Tween 20), the membrane was blocked with 3% (v/v) BSA in TBST buffer overnight at 4°C. The membrane was washed three times for 5 min each time with TBST buffer and incubated with 1% (v/v) BSA in TBS buffer for 2 h at 37°C with specific anti-His-tag antibodies (1:1,000). The membranes were washed three times with TBST buffer and incubated with HRP-conjugated secondary antibodies (Invitrogen, Grand Island, NY, USA) at 37°C for 1 h. After washing three times with TBST buffer, the membrane was analyzed using the DAB substrate kit (Invitrogen).

### Assay of antimicrobial activity

Antimicrobial activity against standard and clinically isolated microorganism strains was analyzed by an inhibition zone assay. The bacteria were grown in LB broth at 37°C. A bacterial dilution (100 *μ*l; OD_600_=0.5) was taken and added to 6 ml fresh LB broth with 0.8% agar and poured over a 9.0 cm Petri dish, giving an agar depth of 1 mm. When the top agar had hardened, a 10 *μ*l aliquot of test sample filtered through a 0.22-*μ*m filter (Millipore) was dropped onto the surface of the top agar and incubated at 37°C overnight. If the sample examined had antimicrobial activity, a clear zone was formed on the surface of the top agar representing inhibition of bacterial growth. The minimal inhibitory concentration (MIC) was determined in liquid medium according to the method described by Wang *et al* (21). The MIC was determined from three independent experiments performed in triplicate.

### Assay of antifungal activity

The fungi were cultivated on potato/dextrose/agar (PDA) media at 28°C. After 6 days, the non-germinate conidia were inoculated into sterile water. Samples of ∼2×10^4^ cells/ml each of *Alternaria alternata, Penicillium digitatum, Botrytis cinerea, Rhizopus stolonifer*, Penicillium italicum and *Magnaporthe grisea* were seeded in yeast extract peptone dextrone (YPD) media to a final volume of 100 *μ*l, in a flat-bottom 96-well microtiter plate. A two-fold dilution series of cecropinXJ solutions (100, 50, 25, 12.5, 6.25, 3.125, 1.56, 0.78 and 0.39 *μ*M) were added to the plates and kept at 28°C for 24 h. A total of 10 *μ*l 5 mg/ml 3-(4,5-dimethyl- 2-thiazolyl)-2,5-diphenyl-2H-tetrazolium bromide (MTT) solution in PBS (pH 7.4) was added to each well and the plates were incubated for 4 h. Fungal growth was analyzed using a microplate reader at 570 nm. Assays were performed in triplicate for three independent experiments.

### Effect of pH and temperature on cecropinXJ activity

The stability of the purified cecropinXJ at different pHs and temperatures was evaluated. To determine pH and temperature resistance, the purified peptide was incubated at various pH values between 2.0 and 12.0 and temperatures between 4 and 100°C for 1–12 h prior to confirming the antimicrobial activity.

### Hemolysis assays

The hemolysis activity of the peptide was assessed by measuring the release of hemoglobin from human red blood cells as reported previously (22). Serial dilutions of the peptides were used and after incubation for 1 h at 37°C, the cells were centrifuged and the absorbance of the supernatant was measured at 595 nm. Controls for 0% hemolysis (blank) and 100% hemolysis were determined using PBS buffer and 1% Triton X-100, respectively.

### Cell proliferation assay

Cancer cells and 293T cells in the logarithmic growth phase (1×10^5^ cells/ml) were plated independently into a 96-well plate (1×10^4^ cells per well), then incubated at 37°C for 24 h. The media were replaced with 200 *μ*l fresh complete medium containing various concentrations of cecropinXJ and complete medium without cecropinXJ was used as a blank control. After another 24 h, the cells were incubated with 20 *μ*l MTT solution (0.5 mg/ml) and 80 *μ*l culture medium followed by incubation at 37°C for 4 h. Subsequently, 100 *μ*l dimethyl sulfoxide was added to dissolve the formazan crystals formed. The OD of the samples was measured with a spectrophotometer at 570 nm. Experiments were performed at least three times. The cell survival was calculated as follows:

$$\text{Cell}\;\text{viability}\;(\%)={\text{OD}}_{570\;(\text{sample})}/{\text{OD}}_{570\;(\text{control})}\times 100.$$

### Statistical analysis

All measurement results are expressed as mean ± standard error (SE) and performed at least three separate times. The differences between the peptide-treated group and control group were evaluated using the unpaired Student’s t-test using one-way ANOVA by GraphPad Prism 4 software (La Jolla, CA, USA). P<0.05 was considered to indicate a statistically significant result.

## Results

### Expression and purification of recombinant cecropinXJ

**Figure 1:**
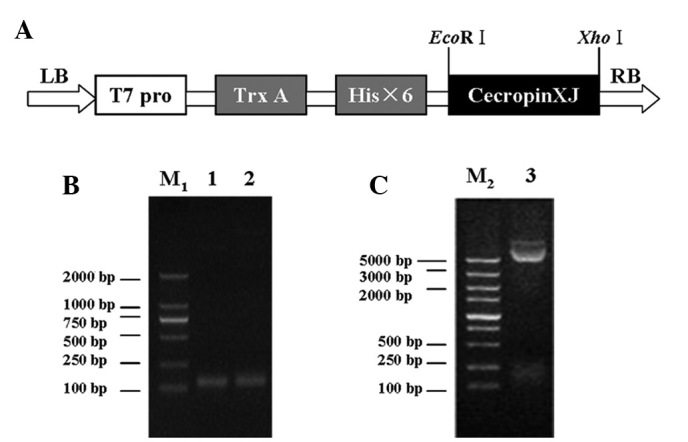
Identification of the recombinant plasmid. (A) Schematic structure of pET32a-*cecropinXJ*. T7 Pro, T7 promoter; LB and RB, left and right border of the expression vector pET32a. (B) Identification of recombinant plasmid by plasmid polymerase chain reaction (PCR). Lane M_1_, DNA molecular weight standards; lane 1, PCR product of the *cecropinXJ* gene fragment with the recombinant plasmid pMD18-T-*cecropinXJ* as template; lane 2, PCR product of the *cecropinXJ* gene fragment with the recombinant plasmid pET32a-*cecropinXJ* as template. (C) Identification of recombinant plasmid pET30a-*cecropinXJ* by digestion with *Eco*RI and *Xho*I. Lane M_2_, DNA molecular weight standards; lane 3, DNA fragment of pET32a-*cecropinXJ* digested with *Eco*RI and *Xho*I. PCR,.

**Figure 2:**
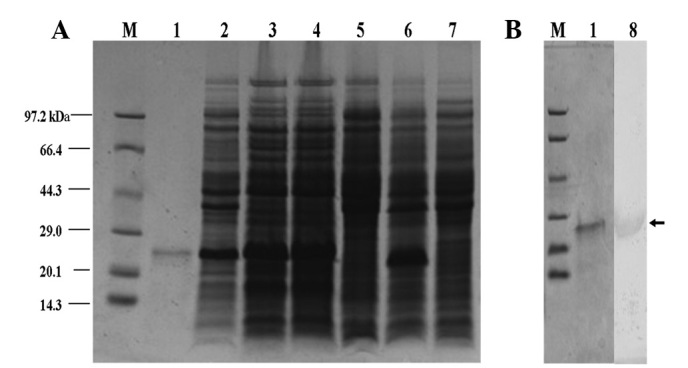
Expression of pET32a-*cecropinXJ* fusion protein analyzed by Tricine-SDS-PAGE. Lane M: Protein molecular mass marker; lane 1: purified pET32a-*cecropinXJ*; lane 2: precipitation of bacterial lysate; lane 3: supernatant of bacterial lysate; lane 4: induced BL21(DE3)-pET32a-*cecropinXJ* (37°C, 0.8 mM IPTG, 5 h); lane 5:uninduced BL21(DE3)-pET32a-*cecropinXJ*; lane 6: induced BL21(DE3)-pET32a; lane 7: induced BL21(DE3); lane 8: western blot of purified pET32a-*cecropinXJ*. IPTG, isopropyl-β-D-thiogalactoside.

The expected molecular masses of 114 and 5700 bp were obtained for the pET32a*-cecropinXJ* plasmid following digestion with *Eco*RI and *Xho*I (Fig. 1). The verified pET32a-*cecropinXJ* plasmid was then transformed into the *E. coli* strain BL21(DE3) that encodes a chromosomal T7 RNA polymerase under the control of a tac promoter. Under IPTG induction, the tac promoter is activated and drives expression of pET32a-*cecropinXJ*. IPTG at a concentration of 0.8 mmol/l efficiently induced the expression of cecropinXJ in the pET32a-*cecropinXJ*-BL21(DE3) system at 37°C, and whole cell proteins were collected for Tricine-SDS-PAGE gel analysis. A major band at the expected size of 25 kDa was observed compared with control as shown in Fig. 2A. The product of recombinant cecropinXJ was mainly present in the supernatant of bacterial lysate following ultrasonic cell lysis and the output was ∼30% of the total bacterial proteins. Purification of recombinant cecropinXJ proteins by Ni-NTA resulted in the isolation of ∼10 mg protein/l of culture (Table I). The specific binding of the His-tag to cecropinXJ was revealed by western blot analysis (Fig. 2B).

### Assay of antimicrobial activity

**Figure 3:**
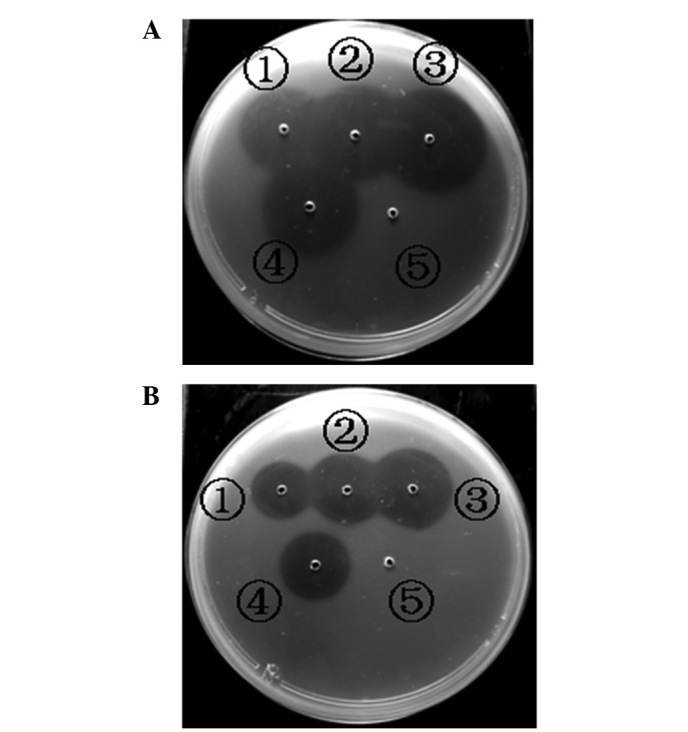
Antimicrobial activity of recombinant cecropinXJ and ampicillin using inhibition zone assays. (A) Gram-positive bacterium *Staphylococcus aureus* treated with (1–3), 1, 2 and 5 *μ*M purified cecropinXJ, respectively; (4), 0.5 *μ*M ampicillin; (5), 10 *μ*l sterile, deionized water. (B) Gram-negative bacterium *Klebsiella penumoniae* treated with (1–3), 1, 2 and 5 *μ*M purified cecropinXJ, respectively; (4), 0.5 *μ*M ampicillin; (5), 10 *μ*l sterile, deionized water.

To confirm that cecropinXJ has an inhibitory effect against several pathogenic bacterial strains, antimicrobial assays were performed as listed in Table II. The purified cecropinXJ showed strong antimicrobial activity against the tested strains (Fig. 3). Of the tested strains, *Staphylococcus aureus* was the most sensitive to cecropinXJ whereas *Acinetobacter baumannii* was not sensitive to this antimicrobial peptide. CecropinXJ inhibited *S. aureus* activity with an MIC of 0.4 *μ*M.

### Assay of antifungal activity

The inhibition zone assay is a simple method for estimating lethal concentrations. However, for certain filamentous fungi, the diameters of the inhibition zones were poorly defined, and for this reason we used a microplate assay to observe the antifungal activity of cecropinXJ under a microscope. As shown in Table III, the MICs of cecropinXJ for *A. alternata, P. digitatum*, *B. cinerea, R. stolonifer*, *P. italicum* and *M. grisea* were 25, 1.56, 6.25, 12.5, 6.25 and 0.78 *μ*M, respectively. CecropinXJ displayed strong antifungal activity against all the tested fungi and fungal growth was completely blocked by cecropinXJ at micromolar concentrations.

### Temperature and pH stability

**Figure 4:**
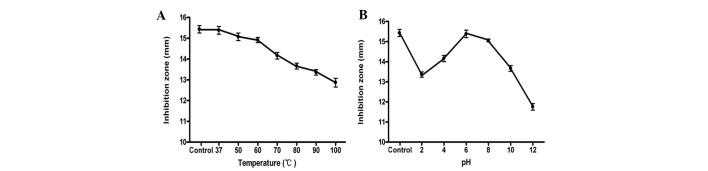
Effects of (A) temperature and (B) pH on cecropinXJ. The controls used were (A) cecropinXJ kept at 4°C and (B) cecropinXJ in the original culture (pH 7). *Staphylococcus aureus* was used as the indicator strain.

The results of the heat stability assay confirmed that cecropinXJ was fully heat stable as the antimicrobial activity of cecropinXJ toward *S. aureus* was retained, even following exposure to 100°C for 12 h (Fig. 4A). CecropinXJ was also observed to be stable at a wide range of pH values as the antimicrobial activity of cecropinXJ was retained between pH 2.0 to 10.0. However, the antimicrobial activity of cecropinXJ was reduced significantly at pH ≥10.0 (Fig. 4B). All assays were performed in duplicate.

### Hemolysis assays

**Figure 5:**
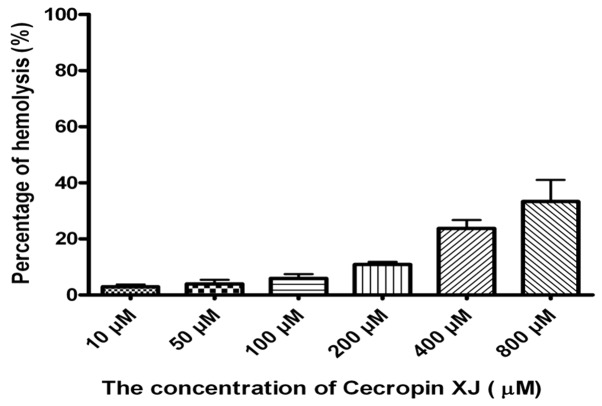
*In vitro* hemolytic activity of cecropinXJ on human erythrocytes. Controls for 0% hemolysis (blank) and 100% hemolysis were determined in phosphate-buffered saline (PBS) and 1% Triton X-100, respectively. Values shown are the means of three independent experiments performed on different occasions with error bars representing standard deviations.

To examine whether cecropinXJ had hemolytic activity, we tested its ability to lyse erythrocytes. CecropinXJ had little hemolytic activity on red blood cells, even at peptide concentrations of up to 200 *μ*M (Fig. 5).

### Cell proliferation and viability assay

**Figure 6:**
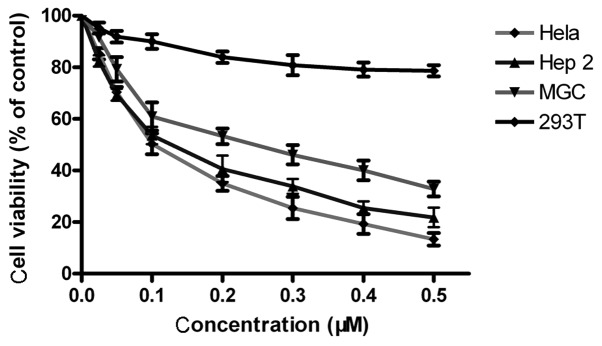
Determination of cell viability by MTT assay. Various cell lines were treated with cecropinXJ in concentrations ranging from 0.01 to 0.5 *μ*M and cell viability was measured by MTT assay. HeLa (human cervical cancer), Hep2 (human laryngeal cancer), MGC (human gastric cancer) and 293T (human embryonic kidney epithelial) cells were cultured in RPMI-1640 medium supplemented with 10% FCS for 24 h at 37°C. Data are expressed as mean ± SEM from three independent experiments.

MTT results showed that cecropinXJ inhibited the proliferation of several cancer cells within 0.01–0.5 *μ*M in a concentration-dependent manner. No inhibition of the proliferation of normal human embryonic kidney epithelial cells was observed (Fig. 6).

## Discussion

AMPs exhibit significant antibacterial activity against Gram-positive and Gram-negative bacteria, and also have potent antitumor activities, which have been studied extensively and are among the most promising candidates for pharmaceutical and antiseptic use (4,7). The difficult isolation and purification processes of the native antimicrobial peptides from their natural sources, as well as the relatively high costs of chemical synthesis, have limited their wide application (23). The ability to produce larger quantities of highly bioactive AMPs at low cost via recombinant DNA technology is important (24). The *E. coli* recombinant expression system is a suitable choice for large-scale production, due to its easy culture, fast growth and effective prevention of bacterial contamination (25). Numerous AMPs have been prepared successfully in *E. coli,* including brevinin-2R (26) and palusterin-2CE (27). A number of fusion partners have been used to express AMPs, including maltose-binding protein, thioredoxin and green fluorescent protein (28–30), to avoid toxicity and proteolysis of AMPs and increase their expression levels in *E. coli* (17,24).

An antimicrobial peptide expressed in bacteria may be cytotoxic to the host or subjected to degradation by host-derived peptidases (24,31). To overcome these potential problems, we fused the DNA coding sequence of a cecropinXJ gene analog within the sequence of a bacterial thioredoxin gene, which was expressed in the pET32a(+) expression system under optimized conditions (15,32).

In a previous study, culture and induction conditions were demonstrated to affect the expression of the soluble target proteins (24). The highest level of soluble expression was achieved at 37°C and 0.8 mM IPTG with a yield reaching 30–35% of the total bacterial proteins (Fig. 1), which was higher than for other AMPs, including adenoregulin (33), perinerin (34), brevinin-2R (26) and ranalexin (35), which had yields of 20, 20–25, 25 and 23–28%, respectively. Subsequently, purification was relatively simple and efficient for preparing large quantities of fusion proteins by affinity chromatography. The yield of cecropinXJ fusion protein reached 10 mg/l of bacterial culture. By comparison of the expression of cecropinXJ with that of other cecropin peptides (36–39), the fusion technology may be employed as a new method for the production of recombinant cecropin peptides.

The recombinant cecropinXJ demonstrated potent antimicrobial activity and a broad antimicrobial spectrum against Gram-positive and Gram-negative bacteria (Table II) and against fungi (Table III). In addition, cecropinXJ retained a high stability against *S. aureus* under different temperatures ranging from 4 to 100°C and pH values ranging from pH 3.0 to 12.0 (Fig. 4). Earlier studies had indicated that cecropinXJ shares a similar structure with ABP-CM4, which has the ability to form specific amphipathic α-helices which allows targeting of nonpolar lipid cell membranes. Upon membrane targeting, the helices form ion-permeable channels, subsequently resulting in cell depolarization, irreversible cytolysis and finally cell death (40–42).

A previous study observed that short cationic peptides consisting of arginine, leucine and lysine inhibit the proliferation of certain types of tumor cells, but do not affect normal cells (43). Differences between the membranes of tumor cells and normal cells contribute to the selectivity of antimicrobial peptides for tumor cells (44–46). In addition, the amphipathic helix of cecropinXJ may play an important role in killing cancer cells by causing leakage of the biomembranes and cytoskeleton. Due to their selectivity, this type of peptide is a good candidate for development as an antitumor agent.

In summary, we provided a simple, economical strategy for producing the antimicrobial peptide cecropinXJ without affecting its antimicrobial activity. Recombinant antibacterial peptide cecropinXJ shows potent activities against bacteria, fungi and tumor cells, and may be a promising candidate for use as a new antibiotic. Furthermore, this study provides a basis for the pharmaceutical application of cecropinXJ.

## Figures and Tables

**Table I:** Isolation of recombinant cecropinXJ based on 1 l of bacterial culture.

Purification step	Total protein (g)	TrxA-*cecropinXJ* (g)	Purity (%)^c^
Sonicated supernatant	1.147±0.614^a^	0.998±0.215^b^	Not applicable
Ammonium sulfate precipitation	0.715±0.347^a^	0.429±0.208^b^	>60
HisTrap	0.016±0.003^a^	0.015±0.002^c^	>90

The wet weight of cells was 4.754±0.681 g.

a Protein concentration was determined by Bradford protein assay.

b Percentage of fusion protein (TrxA-*cecropinXJ*) from total proteins was estimated by Tricine-SDS-PAGE gel scanning.

c Purity of protein was estimated by staining Tricine-SDS-PAGE gel with Coomassie blue.

**Table II:** Antimicrobial activity of cecropinXJ.

Microorganisms	MIC (*μ*M)^a^
Gram-negative bacteria	
*Klebsiella pneumoniae*	0.8
*Shigella flexneri*	2.4
*Acinetobacter baumannii*	4.8
*Shigella sonnei*	2.4
*Klebsiella ozaenae*	3.6
Gram-positive bacteria	
*Staphylococcus aureus*	0.4
*Enterococcus faecalis*	1.2
*Staphylococcus epidermidis*	2.4

a These concentrations represent mean values of three independent experiments performed in duplicate. MIC, minimum inhibitory concentration.

**Table III:** Antifungal activity of cecropinXJ.

Fungus	MIC (*μ*M)^a^
*Alternaria alternata*	25
*Penicillium digitatum*	1.56
*Botrytis cinerea*	6.25
*Rhizopus stolonifer*	12.5
*Penicillium italicum*	6.25
*Magnaporthe grisea*	0.78

a These concentrations represent mean values of three independent experiments performed in duplicate. MIC, minimum inhibitory concentration.
